# High-Temperature Properties and Applications of Si-Based Polymer-Derived Ceramics: A Review

**DOI:** 10.3390/ma14030614

**Published:** 2021-01-29

**Authors:** Zhongkan Ren, Shakir Bin Mujib, Gurpreet Singh

**Affiliations:** Department of Mechanical and Nuclear Engineering, Kansas State University, Manhattan, KS 66506, USA; zhongkan@ksu.edu (Z.R.); sbmujib@ksu.edu (S.B.M.)

**Keywords:** high-temperature, polymer-derived ceramic, fibers, ceramic matrix composites, microelectromechanical systems sensors

## Abstract

Ceramics derived from organic polymer precursors, which have exceptional mechanical and chemical properties that are stable up to temperatures slightly below 2000 °C, are referred to as polymer-derived ceramics (PDCs). These molecularly designed amorphous ceramics have the same high mechanical and chemical properties as conventional powder-based ceramics, but they also demonstrate improved oxidation resistance and creep resistance and low pyrolysis temperature. Since the early 1970s, PDCs have attracted widespread attention due to their unique microstructures, and the benefits of polymeric precursors for advanced manufacturing techniques. Depending on various doping elements, molecular configurations, and microstructures, PDCs may also be beneficial for electrochemical applications at elevated temperatures that exceed the applicability of other materials. However, the microstructural evolution, or the conversion, segregation, and decomposition of amorphous nanodomain structures, decreases the reliability of PDC products at temperatures above 1400 °C. This review investigates structure-related properties of PDC products at elevated temperatures close to or higher than 1000 °C, including manufacturing production, and challenges of high-temperature PDCs. Analysis and future outlook of high-temperature structural and electrical applications, such as fibers, ceramic matrix composites (CMCs), microelectromechanical systems (MEMSs), and sensors, within high-temperature regimes are also discussed.

## 1. Introduction

Ceramics, which are often defined as nonmetallic, inorganic solid materials, have existed for more than 9000 years [1]. Wide varieties of ceramic compounds yield various bonding types (e.g., covalent, metallic, ionic, or mixed) and adjustable physical, and chemical properties [2,3]. Since the early 21st century, high-temperature structural ceramics have gained popularity due to their low density, high oxidation and chemical resistance, outstanding creep resistance, and thermal shock resistance as structural materials [4].

Conventional Si-based advanced ceramics such as SiC or Si_3_N_4_, which are manufactured via the powder route [5], have shown exceptional creep and oxidation resistance up to temperatures exceeding 1000 °C without loss of structure and functionality. However, the extremely high processing temperature of conventional ceramics complicates the production of complex-shaped ceramics, and the brittle nature of ceramics prohibits casting or machining. Although the development of additive manufacturing technologies means that powders can generate complex structures as easily as metals or polymers [6], energy-efficient fabrication of fibers, coatings, films, or ceramic matrix composites (CMCs) from powders is still difficult [5].

**Figure 1:**
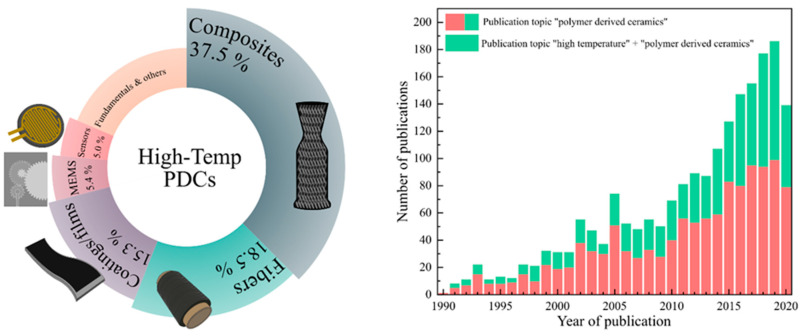
High-temperature-polymer-derived ceramics (PDCs) for different applications (**left**). Publication topics on the keywords “polymer derived ceramics” and “high temperature + polymer derived ceramics” (**right**). Data obtained from 1990 to September 2020 on from ISI web of science.

**Figure 2:**
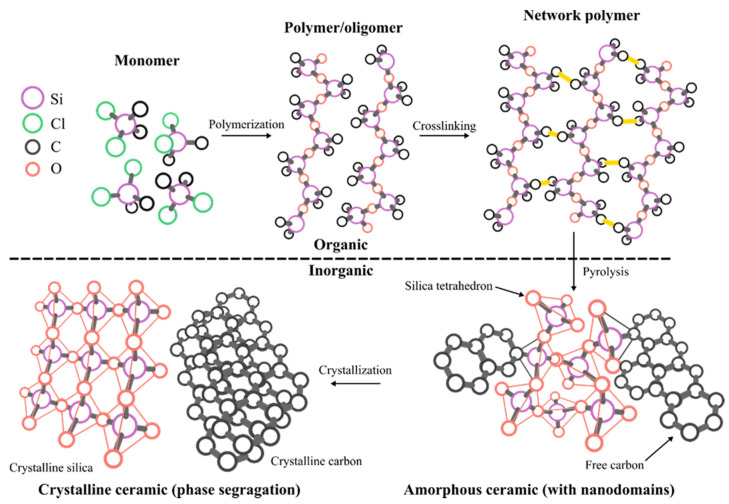
Schematics of polymer-derived ceramic processing route. The shaping of the preceramic polymer occurs at the crosslinking stage by thermal crosslinking. Organic to inorganic transition is completed at the thermal pyrolysis stage at elevated temperatures around 1000 °C.

The polymer-derived ceramic (PDC) approach is an advanced ceramic manufacturing technique that designs or controls molecular structures of ceramic products at molecular or atomic levels [3]. There has been a rapid increase in PDC-related research, as well as PDCs for high-temperature applications, over last 30 years (Figure 1). PDCs are unique because amorphous ceramics are not achievable via other ceramic synthesis techniques (e.g., powder sintering or chemical vapor deposition) [7]. Polymeric network structures become amorphous microstructures during pyrolysis and form nanodomains of 1–3 nm, which provide good creep and oxidation resistance up to 1500 °C. Thereby, thermodynamically unstable ternary ceramics (e.g., SiCN, SiOC, and BCN) and quaternary ceramics (e.g., SiOCN, SiBCN, and SiAlOC) are achieved and exist as stable microstructures (e.g., free C, SiO_2_, Si_3_N_4_, SiC, BN, Al_2_O_3_). The detailed PDC synthesis processes are presented in Figure 2.

**Figure 3:**
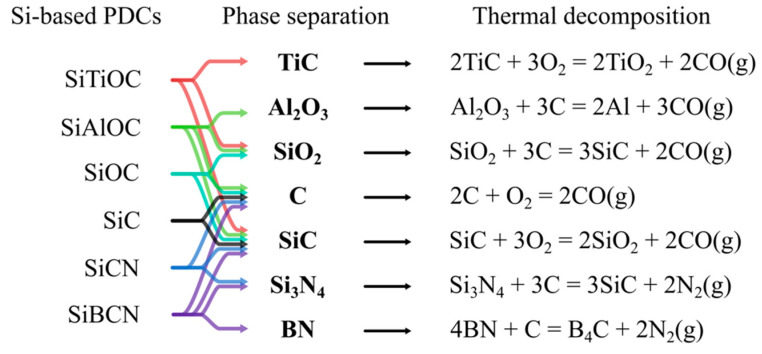
Schematics of phase separation and thermal decomposition of Si-based PDCs at elevated temperatures.

Silicon-based PDCs are produced via thermal pyrolysis of crosslinked organosilicon precursors that may contain light elements such as C, H, O, N, or B at temperatures as high as 1400 °C [8]. Depending on the specific ceramic elemental configuration, some PDCs have high-temperature applications such as coatings, reinforcement, sensors, or matrices [9]. SiCN amorphous ceramics are generally produced from preceramic precursors that contain Si, N, and C via high-temperature (usually ~1000 °C) annealing in inert environments. High-temperature (usually >1100 °C) environments cause the crystallization of amorphous SiCN with microstructures of mixed SiN_4−x_C_x_ silicon tetrahedrons [9] and segregation into thermodynamically stable crystalline microstructures (e.g., SiC, Si_3_N_4_, C, or Si) at higher temperatures [8]. However, excessive carbon leads to the decomposition of ceramic structures at temperatures above 1500 °C (as shown in Figure 3). SiOC PDCs have amorphous microstructures of mixed SiO_4−x_C_x_ that separate into SiO_2_, SiC and C at high temperatures and ultimately degrade into SiC and CO gas (>1300 °C).

Incorporating other elements, such as B [8,10,11,12], Al [13,14,15], Nb [16], Zr [4,17,18], Ti [19,20,21], or Hf [12,22,23,24], in the Si-C-N or Si-O-C systems may greatly enhance the thermostability of ceramic products by preventing or impeding the crystallization and phase segregation of the ceramic phases in inert or oxidizing environments [7]. Initially, at the crosslinking stage, B atoms may improve the formation of the crosslinking network and increase the yield and structural density of SiBOC [11,12]. Upon polymer-to-ceramic conversion, doped elements may form additional phases (e.g., TiO_2_, HfO_2_, ZrO_2_) and lead to nanocomposite-like structures of metal-oxide/amorphous ceramics [25] or dissolve into and alter the amorphous phase (e.g., SiBOC, SiAlOC) which eventually segregate into additional phases (BN, Al_2_O_3_) at higher temperatures. At the amorphous stage, the superior stability of the B- or Al-doped amorphous phase significantly enhances the oxidation resistance of SiBOC or SiAlOC ceramics [11,13]. For example, SiAlOC may stay amorphous at much higher temperatures (up to 1400–1500 °C) compared to SiOC phase (crystallize at ~1300 °C) [14]. The doped SiOC phase has been shown to impede the diffusion of O_2_ at high temperatures and inhibit the decomposition of the microstructure [11,15].

After forming additional microstructures, the presence of TiO_2_, HfO_2_ or ZrO_2_ in the amorphous PDC matrix prevent the crystallization and decomposition of the amorphous phase [18,21,24]. Recent work has shown that presence of TiO_2_ nanocrystals improves the viscosity of the PDC microstructures at elevated temperatures which deters collapse in the TiO_2_/SiO_2_ structure [21].

As previous studies have shown, the phase, microstructures, thermal stability, and high-temperature behaviors of synthesized ceramics can be modified by incorporating different types of hetero-elements into the Si-based precursors at the molecular level. Figure 3 shows the phase separation and thermal decomposition of various Si-based PDCs at elevated temperatures.

Further information on the processing/manufacturing [6,26,27,28,29,30,31,32], physical and mechanical properties [26,27,28,29,30,31,33,34,35,36,37] and micro/nanostructures [5,26,27,29,31,32,34,35,38,39,40] of PDCs can be found in the previously published review articles.

## 2. Applications

### 2.1. Fibers and Matrices

PDC fibers are popular PDC products due to the relentless demand for high-temperature, ceramic-matrix aerospace components [7]. Reinforcing PDC fibers provides strength and structural foundation or shape to the composite, generally in the form of a complex 3D woven structure designed to closely match the final shape of the component [41].

**Figure 4:**
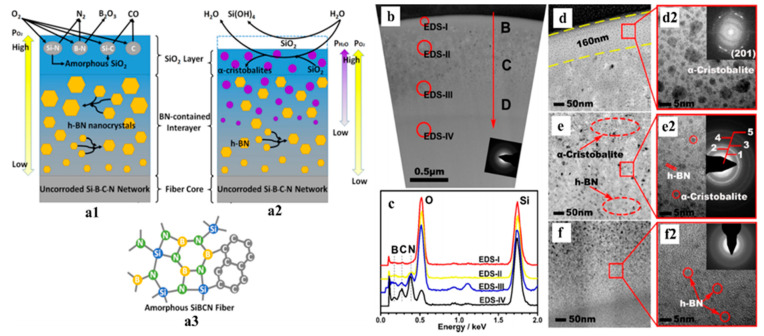
(**a**) Schematics of SiBCN fiber microstructures (**a3**) with a detailed illustration of the formation of two additional surface layers after annealing in (**a1**) air and (**a2**) imitated combustion environment. (**b**,**d**–**f**) Transmission electron microscopy (TEM) images of fiber cross-section showing different layers. (**c**) EDS data obtained from the marked area in (**b**). Reproduced with permission [42]. Copyright 2018, American Chemical Society.

Ji et al. investigated high-temperature antioxidation of amorphous SiBCN fibers, derived from polyborosilazane for aerospace applications [42]. Initial structural characterization suggested amorphous fiber structures of silicon-nitride tetrahedrons (SN_3_C, SiN_4_), free carbon, BN hexatomic rings, and BN_2_C phases (Figure 4a). Crack-free fibers were exposed to air and an imitated combustion environment (with H_2_O, O_2_, and N_2_) at 1400 °C for 2 h. Although subsequent structural investigations indicated well-preserved fiber morphologies with crack-free surfaces and interiors with negligible compositional changes, microstructural changes were captured. Two extra layers formed at the surface of the fibers due to phase separation in which the outermost layer was the SiO_2_ phase, the intermediate layer consisted primarily of the hexagonal BN phase, and the core was the unaffected amorphous SiBCN structure. The combustion environment produced more crystalline SiO_2_ in the amorphous SiO_2_ surface layer than the samples annealed in the air, possibly due to the presence of H_2_O molecules.

**Figure 5:**
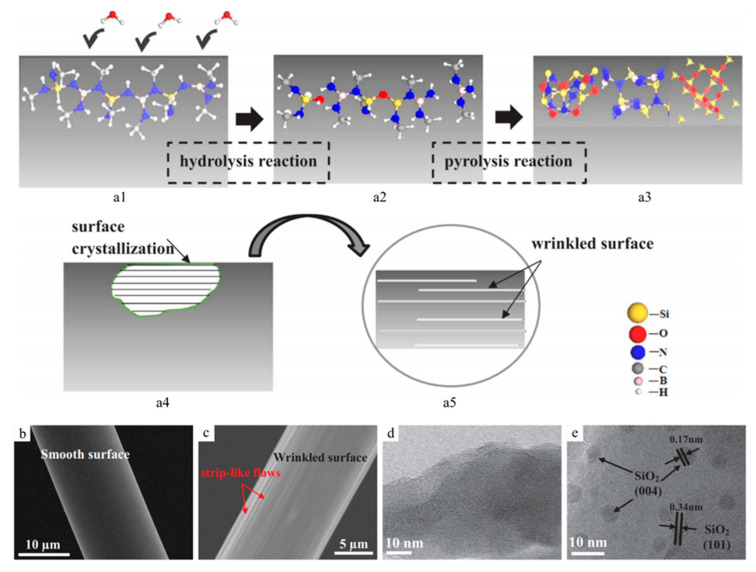
(**a**) Schematics of fiber hydrolysis and pyrolysis reaction. Scanning electron microscopy (SEM) images of pyrolyzed ceramic fibers from (**b**) preserved green fibers in an inert environment and (**c**) exposed green fibers to a humid atmosphere. TEM images of pyrolyzed ceramic fibers from (**d**) preserved green fibers and (**e**) exposed green fibers. Reproduced with permission [43]. Copyright 2018, Elsevier.

As mentioned, excessive carbon in PDC structures often causes reduced thermal stability of the system due to direct oxidation in air or a reaction with other phases to form crystalline structures. Recent studies on a novel ternary system with only Si, B and N have shown an applicable synthesis of Si-based, carbon-free ceramic fibers from polyborosilanes. Specifically, the ceramics were synthesized from precursors without carbon. For example, Cl_3_Si-NH-BCl_2_ can crosslink into oligomers or polymers via polycondensation in NH_3_, and additional pyrolysis produces C-free SiBN ceramics, which have theoretical thermal stability up to 1700 °C. However, this type of precursor requires handling in a dry inert atmosphere due to hydrolysis reactions of the precursors with moisture. Liu et al. studied the hydrolysis effect of polyborosilazane on SiBN ceramic fibers, demonstrating evidence of the formation of Si-O-Si groups in the precursor (Figure 5) [43]. The incorporated oxygen altered the surface features of pyrolyzed PDC fibers and caused the formation of β-SiO_2_ at 1400 °C, leading to decreased thermal stability due to early crystallization.

In the 1990s, SiC/SiC composites were manufactured for high-temperature structural applications based on the highly thermal resistant SiC fibers [44]. Composites of ceramic matrices are known as ceramic matrix composites (CMCs). In general, four industrial techniques have been developed to produce matrices on preshaped woven fiber parts: chemical vapor infiltration (CVI), polymer impregnation and pyrolysis (PIP), reactive melt infiltration (RMI), and slurry infiltration and hot pressing (SI-HP) [45]. The PIP technique utilizes PDC routes with handling and processing advantages, such as low manufacturing temperature and pressure [46].

**Figure 6:**
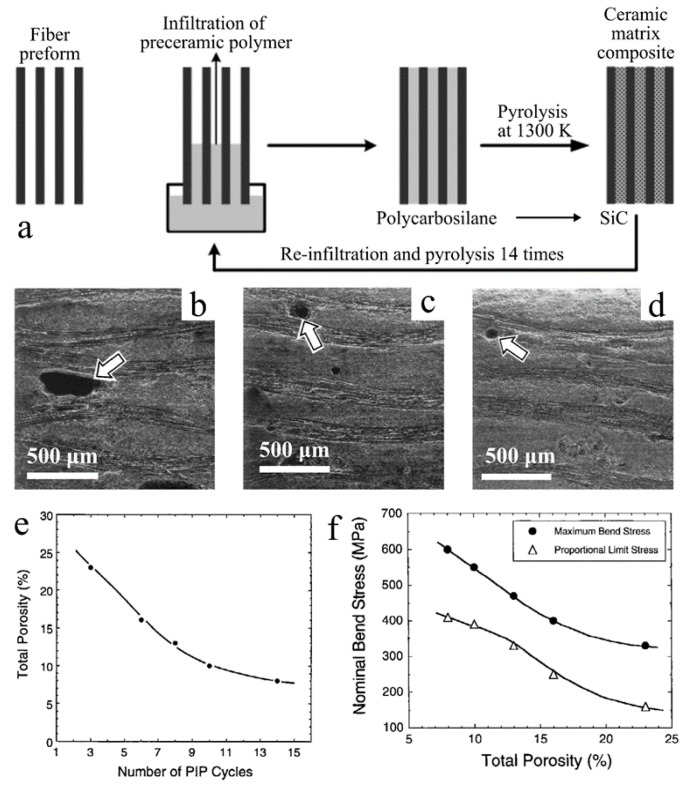
(**a**) Schematics of the polymer impregnation and pyrolysis (PIP) process [48]. SEM images of cross-sectional view of SiC/SiC composite after PIP cycle of (**b**) 3, (**c**) 8 and (**d**) 14. Plots of (**e**) total porosity vs. the number of PIP cycles, and (**f**) nominal bending stress vs. total porosity. Reproduced with permission [44]. Copyright 2004, John Wiley and Sons.

PIP-fabricated SiC matrices have up to 30% porosity, which reduces the strength of the composite product [44]. Infiltration is often necessary to minimize residual porosity of the ceramic matrix phase during the PIP process. Takeda et al. determined the effect of infiltration on total porosity and bending strength of PIP-fabricated SiC/SiC composites (Figure 6) [44]. The results showed that the porosities of SiC CMCs were decreased from 23% to 10% after 14 PIP cycles, and nominal bending strength had an approximately 50% increase from less than 400 to 600 MPa. Yang et al. investigated the thermal shock properties of a 3D woven carbon fiber (C_f_)-reinforced PIP prepared SiC composite [47]. The thermal quench of the specimens was performed from 1500 °C in the air to 100 °C boiling water with a holding time of only 10 min between each environment. Thermal degradation (approximately 13% weight loss) of CMC and a significant decrease in specimen strength were observed after five cycles. Comparatively, the CVI-SiC-coated 3D C_f_-reinforced SiC composite showed over 99% mass retention and less of a decrease in tensile strength under the same condition.

The PIP technique also allows the production of ternary ceramics, such as SiCN or SiOC. Mainzer et al. investigated the thermal stability of C and SiC fiber-reinforced SiCN CMCs derived from low viscous poly(methyl vinyl)silazane pyrolyzed at 1300 °C for 8 PIP cycles [49]. Two commercial fibers for each SiC and C_f_ were selected as the reinforcement for CMCs. The prepared specimen had a maximum mechanical performance of 478 MPa for tensile strength and 206 GPa for modulus. However, after exposing the samples to air at 1200 °C for 10 h, CMCs showed a significant decrease in tensile strength up to 43% for SiC-reinforced samples, and 98.6% for C_f_-reinforced CMC. This expected phenomenon was due to oxidation of the fiber–matrix interface in which the cracked interface provided an oxygen-diffusion path and caused strong oxidation in the SiCN/SiCN interfaces. When the fibers were precoated with CVD-SiC, however, the tensile strength retention reached 89% under the same oxidation environment.

**Figure 7:**
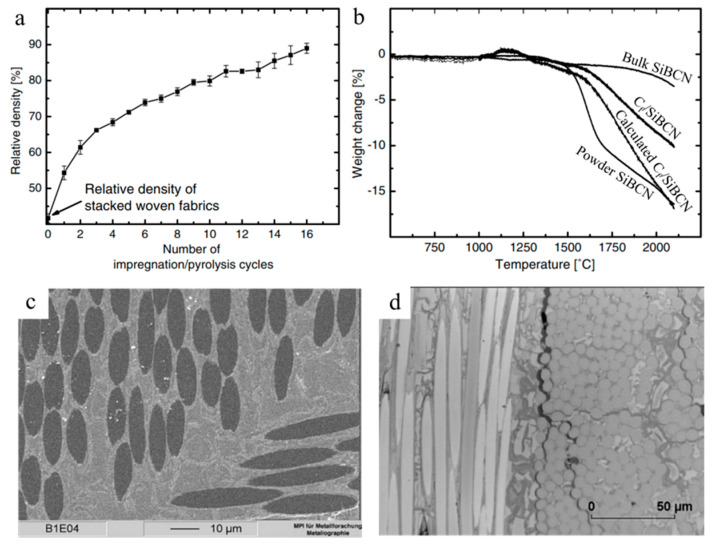
(**a**) Plot of relative density vs. number of impregnation cycles. (**b**) TGA curves of bulk and powdered SiBCN, C_f_/SiBCN composite and calculated C_f_/SiBCN. SEM images of (**c**) as-prepared crack-free interface, (**d**) crack propagated interface [50]. Copyright 2008, Elsevier.

Lee et al. reported enhanced thermal stability, creep resistance and flexural strength of C_f_-reinforced SiBCN composites compared to other matrices, such as SiOC (Figure 7) [50]. In a total of 16 cycles, polyacrylonitrile (PAN)-derived carbon fiber mats were infiltrated with preceramic polymers that converted to SiBCN after pyrolysis at 1400 °C. The resulting CMC reach 89% of relative density with crack-free interfaces. The in situ four-point bending test at 1500 °C in Ar suggested bending flexural strength up to 255 MPa without brittle fracture. Creep testing at 1400 °C for 60 h with 100 MPa pressure in Ar showed a total creep strain of 0.55%, and thermal stability testing showed that thermal degradation of the SiBCN matrix began at approximately 1500 °C. An additional increase in environment temperature drastically decreased flexural strength to 70 MPa at 1700 °C, and 43 MPa at 2000 °C.

### 2.2. Microelectromechanical Systems and Semiconductors

In the early 1990s, Mocaer et al. initially measured the electrical conductivity of SiCNO at a temperature range up to 500 °C, showing a semiconducting feature of PDCs [51]. Later, Ryu et al. measured and confirmed the semiconductive properties of SiCNO PDCs up to 1300 °C without dopants [51]. The band-gap change measured for SiCNO PDCs varied from 2.2 to 0.1 eV as the annealing temperature rose from 1100 to 1400 °C. The results also suggested tweakable semiconductive properties of PDCs by tailoring the elemental compositions. This semiconducting feature of PDCs have founded a new variety of high-temperature semiconducting field and is appealing to other applications, such as sensing or catalyzing. Further electrical characterization of SiCNO PDCs by Terauds et al. exposed significant temperature-dependent piezoresistivity at temperatures up to 1000 °C [52]. Their research identified the dominant (one order of magnitude higher) gauge factors of this PDC over other commercial materials, such as Ge, Si, SiC, or diamond at higher temperature limits, providing an efficient alternative material to high-temperature stress sensors. However, all the electrical properties are manipulatable by controlling the microstructures [53].

**Figure 8:**
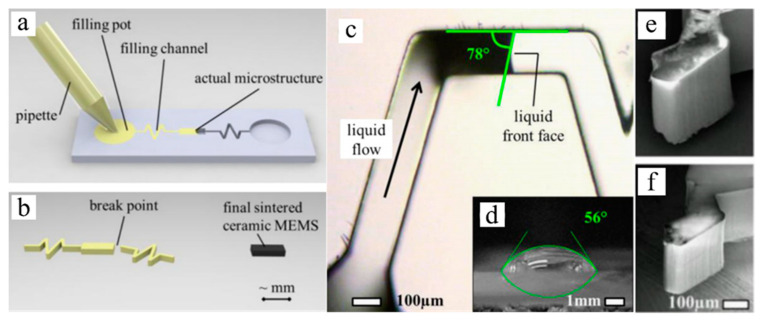
Schematics of shaping process of precursor for microelectromechanical systems (MEMSs), (**a**) mold filling, and (**b**) crosslinking and pyrolysis of samples. Optical microscopy of (**c**) mold filling process, (**d**) precursor drop on the mold surface. SEM images of (**e**) crosslinked sample and (**f**) pyrolyzed sample. Reproduced with permission [56]. Copyright 2008, Elsevier.

As PDC fibers and composites began to be utilized as critical components of advanced applications (e.g., turbine engine for aerospace purposes) in high-temperature environments, the simultaneous controlling and monitoring of high-temperature and harsh working environments became essential [54]. Silicon-based MEMSs have been used in applications such as sensors or actuators, but have demonstrated limited performance due to poor thermal and mechanical stabilities at temperatures above 250 and 600 °C, respectively [54]. SiC, a wide bandgap semiconductor, has also been proposed to overcome working temperature restrictions of Si MEMSs for high-temperature sensing. In addition to an outstanding thermal stability, their superior ant-corrosion, chemical stability and mechanical performance have also attracted wide attention for high-temperature semiconducting applications [54,55]. The shaping process and microscopic features of PDC MEMSs are presented in Figure 8 [56]. However, film-deposition-manufactured SiC is costly to produce and requires extra micromachining, which complicates productions [57]. In comparison, PDC materials have shown great flexibility for MEMS production; the polymer route utilizes micropatterning to allow the shaping of liquid precursors.

**Figure 9:**
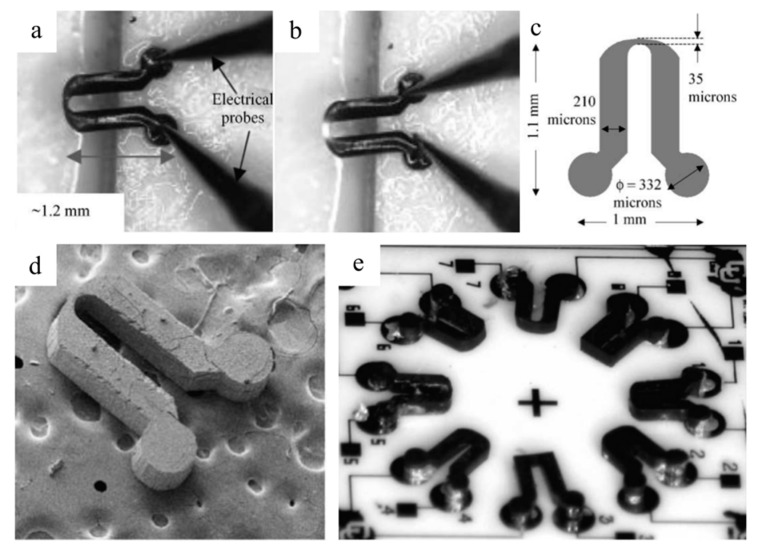
Optical images of SiCN MEMS sensor at (**a**) off state and (**b**) on state. (**c**) Schematics of the design of SiCN glow plug. (**d**) SEM image of SiCN plug. (**e**) Design and packed device image of the sensor part. Reproduced with permission [58]. Copyright 2003, Elsevier.

In the early 2000s, Liew et al. proposed SiCN ceramic microparts with a feature size of 20 microns for MEMSs derived from an injectable liquid precursor (Figure 9) [58]. The produced microparts showed approximately 150% and 66% improved strength and hardness, respectively, over powder-based PDC samples. The manufactured parts bonded without interface using the same liquid precursor as an adhesive via second crosslinking.

### 2.3. Membranes, Coatings and Adhesives

**Figure 10:**
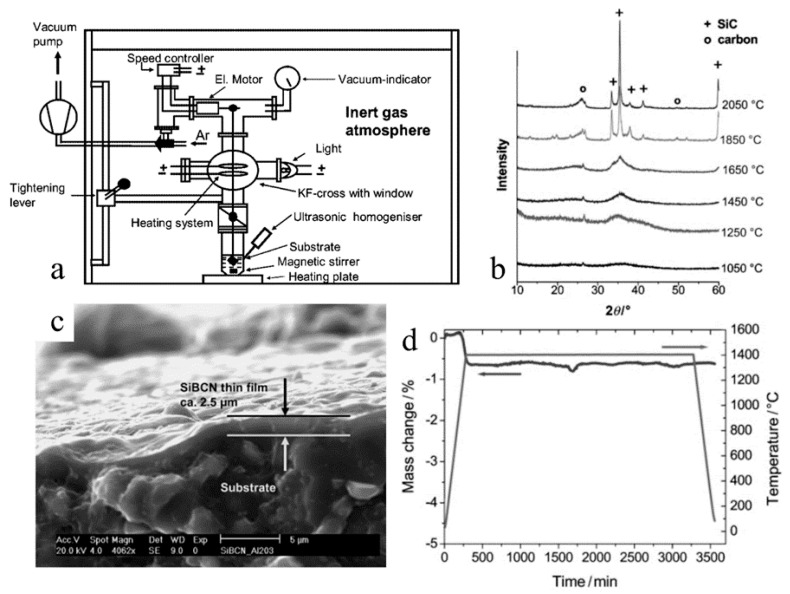
(**a**) Schematics of coating synthesis setup. (**b**) XRD of SiBCN PDC at different annealing temperatures, suggesting conversion of amorphous to crystalline at 1850 °C. (**c**) SEM images of the cross-section of SiBCN membrane on an alumina substrate. (**d**) TGA of SiBCN at 1400 °C for 50 h in the air [10].

SiBCN is a promising high-temperature PDC material that has shown significant advantages over commercial alumina membranes, including ease of manufacturing via a sol-gel process and high thermal stability and resistance to oxidation and phase transformation [10]. Hauser et al. showed the potential of SiBCN as high-temperature separating membranes (Figure 10) [10]. They synthesized ceramic film on an alumina substrate surface by dip-coating the substates into the precursor solution. A thin SiBCN film with a thickness of 2.5 microns was produced on porous alumina, with pore sizes of 0.6–6 nm. This SiBCN, which was PDC-derived from a cyclic preceramic precursor and borane dimethylsulfide, showed high thermal stability at 1400 °C in an oxidizing environment with a mass drop of only 1 wt.% for 50 h, free of crystallization.

PDC ceramic coatings have also gained attention because, similar to membranes, they are easily manufacturable on large complex surfaces with fine structural and thermal performances at energy efficient pyrolysis temperatures as low as 800 °C [59]. Previously, the primary drawback of using PDCs was the coating thickness, which was limited to several microns due to thermal shrinkage upon pyrolysis. Consequently, active or passive filters were added to the system to control volumetric shrinkage and enhance functionalities such as thermal or electrical conductivities, altering thermal expansion and hardness, etc. [59].

**Figure 11:**
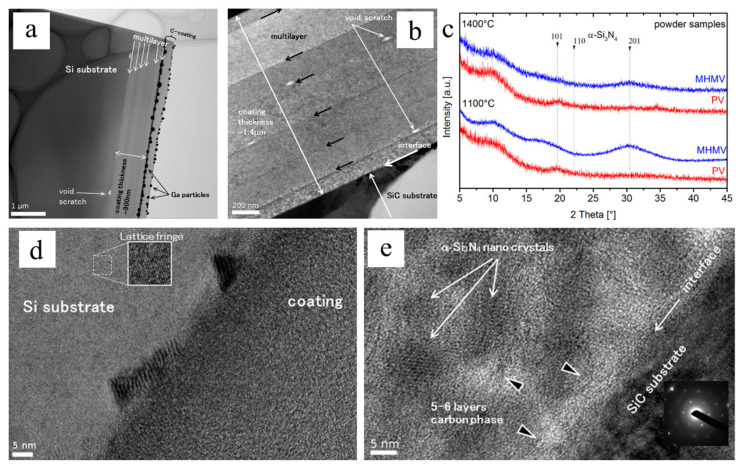
TEM images of (**a**) as-prepared SiCN multilayer coating on Si substrate pyrolyzed at 1100 °C and (**b**) SiCN multilayer coating on SiC substrate annealed at 1400 °C. (**c**) XRD of SiCN as-prepared and annealed samples, both showed amorphous structures. High-res TEM images of (**d**) as-prepared sample on Si substrate and (**e**) annealed sample on SiC substrate. Reproduced with permission [60]. Copyright 2015, Elsevier.

**Figure 12:**
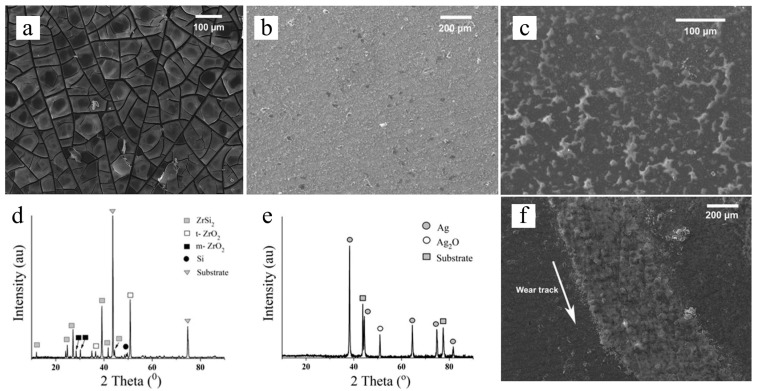
SEM images of (**a**) neat pyrolyzed SiOC ceramic coating with obvious cracks, (**b**) pyrolyzed SiOC-ZrO_2_ coating and (**c**) pyrolyzed SiOC-Ag coating at 700 °C. XRD spectra of (**d**) pyrolyzed SiOC-ZrO_2_ sample and (**e**) pyrolyzed SiOC-Ag sample, where both showed phase oxidation of incorporated particles into ZrO_2_ and Ag_2_O. (**f**) SEM image of wear track of SiOC-ZrO_2_ sample with a sliding distance of 18 m at a load of 3 N. Reproduced with permissions [59]. Copyright 2018, Springer Nature.

Klausmann et al. demonstrated high-temperature structural evolution of one-micron-thick SiCN coatings derived from polysilylcarbodiimide-based linear and branched precursors via spin coating (Figure 11) [60]. Results showed that thermal and mechanical performances are directly related to the carbon content of the PDC: low carbon content showed up to an 80% increase in elastic modulus and hardness at ambient and lower crystallization at annealing of 1400 °C than high-carbon SiCN. A recent study by Alvi et al. investigated the tribological coating performance of SiOC PDCs with ZrSi_2_ and Ag fillers for superior tribological performance at a high load and high temperature (Figure 12) [59]. The characterization suggested well-controlled shrinkage and surface cracks with fillers with a coating thickness of more than 10 microns. X-ray diffraction (XRD) analysis showed the decomposition of ZrSi_2_ filler into ZrO_2_ and possibly SiO_2_ and Si, as well as the oxidation of Ag into Ag_2_O. The formation of ZrO_2_ caused SiOC-ZrSi_2_ coatings to demonstrate better tribological performances with negligible wear and lubricating features in ambient temperatures.

High-temperature adhesives are critical structural materials for aerospace and nuclear power applications. The adhesives must provide decent bonding strength in high-temperature environments. In 2018, Luan et al. conducted an adhesive performance investigation of SiCN for joining Al_2_O_3_ surfaces from polysilaznes with fillers such as polyborosilazane, polysiloxane and Al_2_O_3_ to address shrinkage and improve high-temperature bonding strength. Results showed an approximate 12 MPa bonding strength, obtained from modified adhesive, at room temperature and an approximate 50% decrease at 1000 °C, which was still twice the performance of pure SiCN at the elevated temperature. The coating showed improved thermal stability with nano-Al_2_O_3_ filler, possibly due to mixed bonding between Al_2_O_3_, SiO_2_ and B_2_O_3_ stabilizing the liquefaction of B_2_O_3_ at said temperature range. The research group conducted another investigation of TiB_2_-modified SiCN ceramic adhesive. The formation of SiO_2_, B_2_O, and TiO_2_ during the thermal pyrolysis process resulted in an approximate 10 MPa adhesive strength, which was found at room temperature and 8 MPa at 800 °C. The residual TiB_2_ continued to impede crack propagation.

### 2.4. Sensors

Sensors are often used in advanced systems that require detection and controls. For example, heat flux sensors are mandatory for monitoring gas turbine combustion chamber temperature, pressure and flow [61]. PDC sensors were produced because of their semiconductive behavior; more specifically, the temperature-dependent conductivity feature of PDCs. Silicon, or SiC, sensors have been widely studied and used for temperatures below 500 °C. Novel PDCs may be reliable for high-temperature microsensors. Another potential high-temperature pressure sensor, which is based on the piezoresistivity of doped PDCs, requires the sensing material to withstand the harsh environment at elevated temperatures without losing functionality and structures [62].

**Figure 13:**
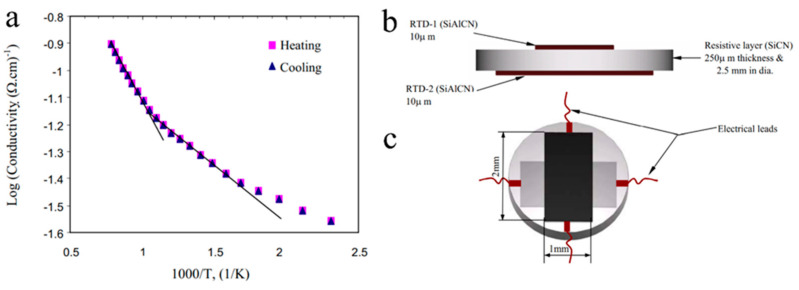
(**a**) Semiconducting behavior of SiAlCN indicated by the changing electric conductivity at different temperatures. Schematics of the design of heat-flux sensor of PDC materials, (**b**) sectional view and (**c**) top view. Reproduced with permission [61]. Copyright 2006, IOP Publishing.

**Figure 14:**
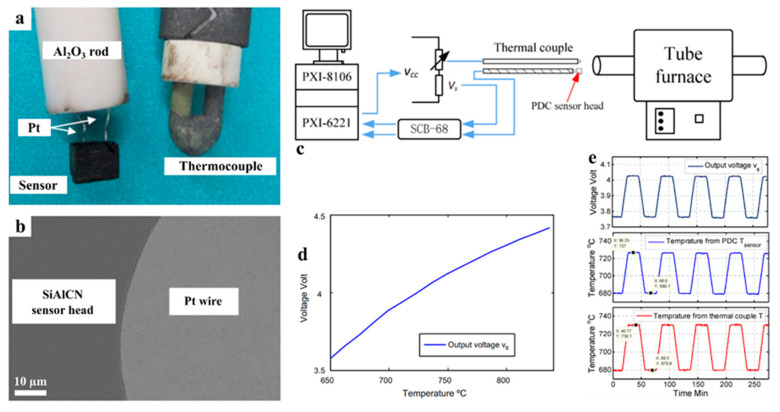
(**a**) Optical images of SiAlCN sensor attached to alumina rod via Pt wires and thermocouple for comparison. (**b**) SEM images of a cross-section of sensor head showing the interface between SiAlCN ceramic phase and Pt wire. (**c**) Schematics of temperature measurement testing setup. (**d**) The output voltage of the temperature sensor as a function of temperature. (**e**) The response of the temperature sensor to the changes of environmental temperature, and the comparison to thermal couple measurement. Reproduced with permission [63]. Copyright 2014, Elsevier.

**Figure 15:**
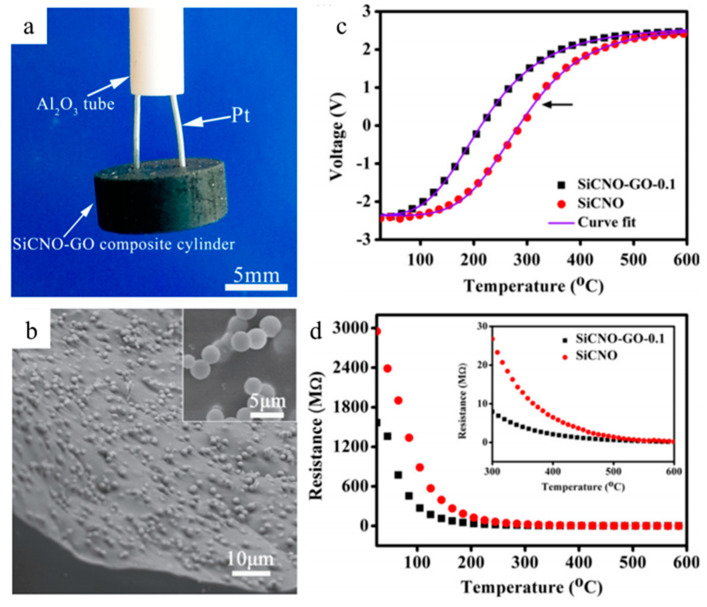
(**a**) Image of SiCNO-GO sensor. (**b**) SEM images of SiCNO-GO (1 wt.% GO) sensor with a magnified image (inset). (**c**) The voltage output of the sensor at the temperature range from 25 to 600 °C. (**d**) The resistance of probe material vs. temperature. Reproduced with permission [64]. Copyright 2016, John Wiley and Sons.

Nagaiah et al. proposed a high-temperature heat flux sensor design with micromachined SiCN as a thermal resistance layer and thin amorphous semiconducting SiAlCN layers as resistance temperature detectors (RTDs), as shown in Figure 13 [61]. Seo et al. then directly tested photolithography manufactured SiCN PDC thin films as RTDs at moderate temperatures, confirming the feasibility of heat flux sensing [53]. Zhao et al. fabricated, tested, and compared a complete temperature sensor with thermocouples using SiAlCN as the probe material (Figure 14). The results showed a repeatable, close-matching result to the thermocouple measurements at temperatures up to 845 °C [63]. In 2016, Yu et al. studied a high-temperature SiCNO sensor derived from polyvinlysilazane without metallic or semiconducting elements, similar to SiAlCN (Figure 15) [64]. SiCNO was functionalized with graphene oxide (GO), which enhanced the electrical conductivity of the ceramic matrix. The composite temperature sensor delivered a more accurate and sensitive measurement of temperature change than the SiCNO temperature sensor.

Based on the giant gauge factors Teraudes et al. found in SiCNO PDCs, Li et al. recently reported a full design and characterization of pressure sensors from Al-doped SiOC PDCs for potential high-temperature usage. The sensor was tested in ambient conditions and showed outstanding accuracy, repeatability and stability with changing pressures.

Another electronic application of PDCs was found as carbon-containing absorbing materials for waves. The use of PDCs resolved issues such as the high density of pure magnetic absorbing materials and the low thermal stability of pure carbon materials. Hou et al. proposed high-temperature oxidation resistive wave-absorbing SiC composites via the PDC route with the incorporation of Fe_3_Si and carbon nanotubes (CNTs) [65]. Experimental results demonstrated the high-temperature antioxidation and tunable wave absorption of SiC/Fe_3_Si/CNTs composite ceramics. The minimal reflection loss (RL) value reached −41.2 dB with a sample measuring 2 mm thick, although the RL value could be −40 dB (at 10.36 GHz with a sample thickness of 3 mm) after heat treatment at 800 °C in the air with mass loss < 4.9%.

High-temperature applications of PDCs are summarized in Table 1.

## 3. Conclusions and Outlook

In this work we highlighted high-temperature properties and corresponding high-temperature applications of PDCs. Specific applications, such as ceramic fibers, fiber-reinforced ceramic matrix composites, MEMSs, semiconductive applications, membranes, coatings, adhesives and sensors, were presented based on mechanical, electrical and thermal performances at elevated temperatures above 500 °C. Combining the outstanding intrinsic properties of ceramics with the unique features of the polymer route, PDCs were shown to have advantages over conventional powder-based ceramics and other types of materials in many fields. For example, PDCs demonstrated superior creep resistance and oxidation resistance compared to conventional powder-based advanced ceramics, as well as more controlled intrinsic porosity, complex shaping capability and lower processing temperatures. PDC-based fiber materials appear to retain high elastic modulus and mechanical strength similar to carbon reinforcement materials (e.g., carbon fibers) even at higher temperatures. PDC based composites offer enhanced toughness, high modulus and strength and increased oxidation resistance at higher working temperatures than traditional single-crystals or superalloys.

In addition, a fine-tuning of desired properties is possible by altering the initial composition of the preceramic polymer and optimizing the temperature and duration of the crosslinking and pyrolysis processes. The incorporation of new elements may also enhance the mechanical and thermal properties, or introduce new properties (e.g., semiconductive behavior) to the PDCs. The use of polymeric precursors allows advanced manufacturing techniques to generate micrometer to submicrometer-sized features, such as micropatterned MEMSs, coatings and nanofibers.

Future advancement of high-temperature PDCs are expected to be in CMCs and advanced manufacturing. Structural CMCs are presently being tested for commercial aerospace engine applications such as turbine components that generally operate in harsh environments; further improvements in mechanical and thermal reliability of the components are needed to ensure the safe use of these next-generation structural materials. On the other hand, advanced manufacturing, especially additive manufacturing (or 3D printing) of PDCs, reported over a decade ago, can now produce submicron feature sizes via laser-assisted techniques (e.g., stereolithography or selective laser sintering). However, sintering or pyrolysis for polymer-to-ceramic conversion often leads to shrinkage, residual porosity and related defects that significantly reduce the mechanical strengths and moduli of the printed components for high-temperature applications.

## Figures and Tables

**Table 1:** Applications, precursors, properties and thermal stabilities of high-temperature PDCs.

Application	PDC	Filler	Precursor	Remarkable Properties	Thermal Stability (°C)	Reference
Fiber	SiCN	-	Polysilazane	D = 100 μm	1400	[66]
	SiBCN	-	Polyborosilazane	σ_T_ = 1.03 GPa	1600 (T_p_)	[67]
	SiBN	-	Polyborosilazane	σ_T_ = 0.91 GPa	1400 (T_p_)	[43]
	SiBCN	-	Polyborosilazane	D = 12 μm	1400	[42]
	SiOC	Pb	Polycarbosilane	WCA = 135°	700	[68]
	SiOC	TiO_2_	Polyhydromethyl-siloxane	WCA = 130°	1300	[69]
	SiOC	HfO_2_	Tetraethoxysilane + dimethyldiethoxyl-silane	σ_T_ = 1.5 GPa	1000 (T_p_)	[70]
	SiOC	HfO_2_	Tetraethoxysilane + dimethyldiethoxyl-silane	σ_T_ = 930 MPa E = 155 GPa	1500	[23]
	SiBOC	-	Polyborosiloxane	D = 1 μm	800	[11]
	SiCN	-	Polysilazane	D < 100 nm	1100 (T_p_)	[71]
Matrix	SiC	-	Polycarbosilane	σ_B_ = 600 MPa	1027 (T_p_)	[44]
	SiC	ZrC	Polycarbosilane	ρ = 2.13 g/cm^3^ porosity = 15%	1700	[72]
	SiC	ZrC	Polycarbosilane	Porosity = 0.9%	1950	[73]
	SiBCN	-	Oligovinylsilazane + tris(methyldihydridosilylethylene)borane	σ_B_ = 225 MPa	1500	[50]
	SiOC	ZrO_2_	Polymethylsilsesquioxane	ρ = 2.3–3.5 g/cm^3^	1400	[4]
Coating/Membrane	SiO_2_	Al	Polyhydridomethylsiloxane	IE = 0.2 in 8–14 μm	800	[74]
	SiOC	Y_2_O_3_	Polysiloxane	CTE = 4.43–7.26 × 10^−6^	1400	[75]
	SiCN	-	Polysilylcarbodiimide	E = 105–117 GPa Hardness = 10–11 GPa	1400	[60]
	SiOC	ZrB_2_	Polycarbosilane	SE_T_ = 72 dB at Ka	1000	[76]
	SiOC	-	Polysiloxane	Conductivity = 11 mW m^−1^ K^−1^	1000	[77]
	SiBCN	-	Polysilazane + borane dimethylsulfide	Ceramic yield = 65%	1800	[10]
Adhesive	SiBCN	Nano Al_2_O_3_	Polyborosilazane + polysiloxane	Bond strength = 6.65 MPa	1000	[78]
	SiBCN	borazine	Polymethylsilane	Bond strength = 7.9 MPa	1600	[79]
	SiCN	TiB_2_	Polysilazane	Bond strength = 8.0 MPa	800	[80]
	SiBCNO	TiB_2_	Polysilazane + polysiloxane	Bond strength = 12.3 MPa	1000	[81]
	SiOC	B_4_C, glass powder	Polymethylsilane + polysiloxane	Bond strength = 66.9 MPa	1000	[82]
	SiOC	-	Polymethylsilane + Polysiloxane	Bond strength = 50.8 MPa	1200	[83]
	SiC	-	Polycarbosilane	Bond strength = 120 MPa	1750	[84]
	SiC	-	Polycarbosilane	Bond strength = 105.8 MPa	1500	[85]
	SiOC	-	Polycarbosilane	Bond strength = 66.9 MPaR = 1.15–5.6 kΩ	850	[86]
MEMS/Semiconductor	SiCN	-	Polysilazane	E = 158 GPa	1500	[87]
	SiCN	-	-	E = 80–225 GPa	1650	[88]
	SiCN	-	Polysilazane	FS = 326 um/s	-	[54]
	SiCNO	-	Polysilazane	BG = 2.2 eV	1300	[51]
Sensor	SiAlOC	-	Aluminum tri-sec-butoxide	E = 150 GPa GF = 16,000	1000	[62]
	SiAlCN	-	-	EC = 0–100/Ω/cm	1400	[61]
	SiCNO	-	Polymethylsilsesquioxane	GF = 600–1700	1500	[52]
	SiCN	-	Polysilazane	R = 60 kΩ	1400	[53]
	SiCN	-	Polysilazane	Sensitivity = 290 kHz kPa^−1^	800	[89]
	SiBCN	-	Polyborosilazane	GF = 5500	1000	[90]
	SiAlCN	-	Polysilzane + aluminum-tri-sec-butoxide	c_1_ = −3235 c_2_ = −3.6	830	[63]
	SiCN	-	Polysilazane	BG = 0.08 eV	1000	[91]
	SiCNO	GO	Polyvinylsilazane	EC = 1.39 × 10^−7^	600	[64]
Wave absorb	SiBCN	-	Polyborosilazane	RL = −12.62 dB at 3.2 GHz	1400	[92]
	SiC	Fe_3_Si, CNTs	Polysilylacetylene + ferric acetylacetonate	RL = −40 dB at 10.36 GHz	800	[65]

T_P_: pyrolysis temperature; WCA: water contact angle; IE: infrared emissivity; CTE: coefficient of thermal expansion; COF: coefficient of friction; SE_T_: electromagnetic interference shielding efficiency; FS: filling speed; BG: bandgap.GF: gauge factor; EC: electrical conductivity; RL: reflection loss.
